# Association between risk-weighted apolipoprotein B and prevalent kidney stones: a retrospective case-control study

**DOI:** 10.3389/fmed.2026.1927930

**Published:** 2026-09-14

**Authors:** Xiaogang Chen, Xin Shen, Mengwei Liu, Yi Liu, Qiankun Zhang, Hao Xu, Xiansheng Zhang

**Affiliations:** 1 Department of Urology, The First Affiliated Hospital of Anhui Medical University, Hefei, Anhui, China; 2 Department of Urology, Huangshi Central Hospital, Affiliated Hospital of Hubei Polytechnic University, Huangshi, Hubei, China; 3 Hubei Key Laboratory of Kidney Disease Pathogenesis and Intervention, Huangshi, Hubei, China; 4 Department of Hepatobiliary, Urologic, and Gynecologic Oncology, Huangshi Central Hospital, Affiliated Hospital of Hubei Polytechnic University, Huangshi, Hubei, China; 5 Department of Urology, Huangshi Central Hospital Graduate Joint Training Base, School of Medicine, Wuhan University of Science and Technology, Huangshi, Hubei, China

**Keywords:** dyslipidemia, kidney stones, multivariable logistic regression, prevalent stone status, risk-weighted apolipoprotein B (RW-apoB)

## Abstract

**Objective:**

To investigate the association between risk-weighted apolipoprotein B (RW-apoB) levels and prevalent kidney stones.

**Methods:**

This retrospective case-control study included 761 subjects, comprising a non-stone group (*n* = 522) and an incident kidney stone group confirmed strictly by non-contrast computed tomography (NCCT) (*n* = 239). Multivariable logistic regression models were utilized to evaluate the association between RW-apoB and prevalent kidney stones. To address unmeasured confounding from potential lipid-lowering therapies, a sensitivity analysis was conducted by excluding individuals with comorbid hypertension and diabetes. Additionally, subgroup analyses were performed to assess the stability of this association across different clinical characteristics.

**Results:**

Baseline analysis revealed that RW-apoB levels in the kidney stone group were significantly higher than those in the non-stone group (95.89 ± 28.80 vs. 88.52 ± 24.42, *p* < 0.001). Multivariable logistic regression analysis indicated that continuously elevated RW-apoB levels were significantly associated with prevalent kidney stones (OR = 1.01, 95% CI: 1.00–1.02). In the strict sensitivity analysis excluding hypertensive and diabetic patients, this positive association remained consistent (OR = 1.01, 95% CI: 1.00–1.03). Furthermore, stratified analysis demonstrated that this positive association between RW-apoB and kidney stones remained relatively consistent across subgroups defined by age, gender, and metabolic comorbidities.

**Conclusions:**

Higher RW-apoB was associated with prevalent kidney-stone status after adjustment for measured clinical covariates.

## 1 Introduction

Nephrolithiasis (kidney stones) is one of the most common and highly prevalent diseases in urology. Extensive recent epidemiological data demonstrate that the global prevalence of kidney stones is continuously rising, and its high incidence and frequent recurrence have imposed a tremendous economic and public health burden on the global healthcare system (1–3). Traditional medical perspectives often attribute kidney stones to dietary habits, local anatomical abnormalities, and alterations in the physicochemical properties of urine (such as pH, oxalate, and calcium concentrations) (4, 5). However, an increasing number of large-scale epidemiological and mechanistic studies in recent years have revealed that kidney stones are not merely an isolated urinary system lesion, but rather a localized pathological manifestation of systemic metabolic disorders within the kidneys (6, 7). Among these, systemic metabolic abnormalities, represented by Metabolic Syndrome, have been confirmed as core risk factors driving stone formation (7, 8). As a crucial link in metabolic disorders, dyslipidemia plays an undeniable role in the occurrence and development of kidney stones (9–11). A hyperlipidemic state can not only induce systemic and local renal oxidative stress and inflammatory responses, leading to lipotoxic injury of renal tubular epithelial cells, but it also alters the urinary excretion of lithogenic factors (such as oxalate, uric acid, and calcium), thereby significantly promoting the nucleation, growth, and adhesion of crystals (12). Therefore, identifying novel clinical biomarkers that can accurately reflect pathogenic lipid disorders and metabolic abnormalities is of great clinical significance for the screening of high-risk populations and early, precise intervention for kidney stones.

Apolipoprotein B (apoB) is the core structural protein constituting all atherogenic lipoprotein particles, including very-low-density lipoprotein (VLDL), intermediate-density lipoprotein (IDL), and low-density lipoprotein (LDL) (13). Because the surface of each aforementioned lipoprotein particle contains only a single apoB molecule, the total concentration of apoB can extremely and accurately reflect the total burden of potentially pathogenic lipoprotein particles in the body (13, 14). In recent years, a large body of authoritative cardiovascular and endocrinological research has confirmed that, compared to traditional low-density lipoprotein cholesterol (LDL-C) or non-high-density lipoprotein cholesterol (non-HDL-C), apoB-based indices possess highly significant superiority in predicting residual lipid risk and metabolic syndrome (13, 15). Currently, apoB-related indices (including the derived RW-apoB, etc.) have been widely and successfully applied in cardiovascular disease risk stratification (16, 17), insulin resistance evaluation (18), and the prognostic assessment of metabolic-associated fatty liver disease (19), and have been strongly recommended by multiple top international guidelines as precise biomarkers for metabolic disorders (16, 17, 20). However, although the pathological mechanism linking dyslipidemia to kidney stones has been confirmed in numerous studies, to date, RW-apoB—an index demonstrating outstanding clinical value in predicting metabolic diseases—has not undergone any clinical exploration in the field of kidney stone risk assessment. In light of this, the present study systematically investigates the association between RW-apoB levels and the prevalence of kidney stones for the first time, aiming to fill the gap in this research field and provide a novel theoretical basis and reference for precision prevention strategies for kidney stones based on metabolic regulation.

## 2 Methods

### 2.1 Study design and participants

This was a single-center, retrospective case-control study, and all data were sourced from the First Affiliated Hospital of Anhui Medical University. The study protocol strictly adhered to the ethical guidelines of the Declaration of Helsinki and was approved by the Ethics Committee of the First Affiliated Hospital of Anhui Medical University. Given the retrospective observational nature of this study, the requirement for patients’ clinical informed consent was waived.

We retrospectively collected data from patients hospitalized in the Department of Urology at the First Affiliated Hospital of Anhui Medical University between August 2024 and March 2026 to serve as the case group (kidney stone group) (Figure 1). Notably, all included case subjects (*n* = 239) were patients presenting with their first episode of kidney stones (incident stones). The inclusion criteria for the case group were: (1) definitive diagnosis of kidney stones confirmed strictly by non-contrast computed tomography (NCCT) imaging; (2) complete clinical and laboratory biochemical data. Exclusion criteria included: (1) co-occurrence of severe acute infection or systemic inflammatory response syndrome; (2) history of hematological malignancies, or severe liver or kidney failure; (3) lack of required laboratory data to evaluate the core exposure variable and key clinical covariates.

**Figure 1:**
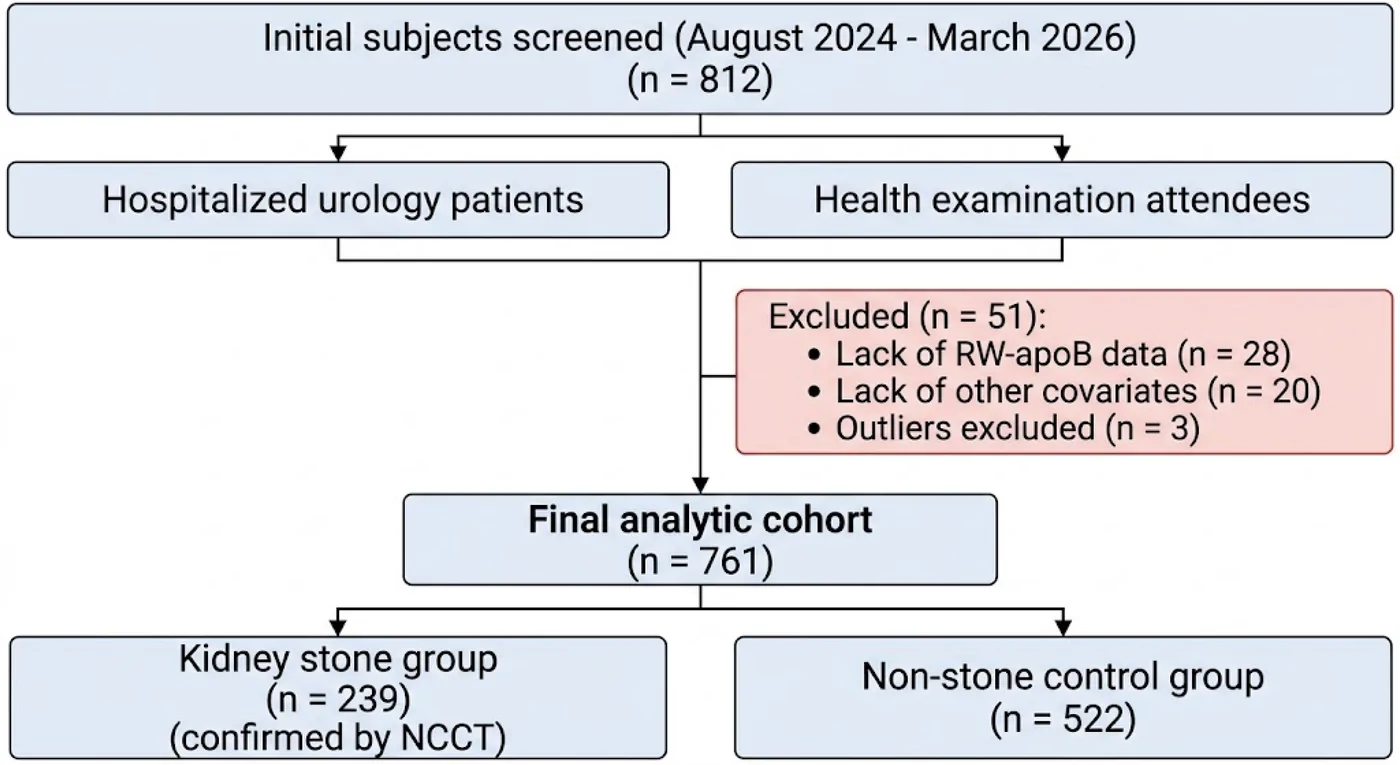
Research flowchart.

Data for the control group (non-stone group) were obtained from a population undergoing routine health examinations at the Health Examination Center of the First Affiliated Hospital of Anhui Medical University during the same period. The inclusion criteria for the control group were: (1) definitive exclusion of kidney stones and other urinary system space-occupying lesions via imaging modalities such as urinary ultrasound; (2) complete clinical and laboratory biochemical data for the same period. Ultimately, this study included a total of 761 eligible subjects, comprising 239 patients with NCCT-confirmed incident kidney stones (case group) and 522 non-stone individuals undergoing concurrent health examinations (control group). For clinical data assessment, fasting venous blood samples for all participants were collected in the morning following an overnight fast of at least 8 h. For hospitalized cases, blood samples were drawn on the morning following admission and prior to any surgical intervention, whereas for controls, samples were drawn during their morning health examination.

### 2.2 Data collection and variable definitions

All baseline demographic data and clinical biochemical indicators were extracted through the hospital's electronic medical record system at the time of patient admission or routine physical examination. The collected baseline covariates included: Age, Gender, Body Mass Index (BMI, calculated as weight (kg)/height^2^ (m^2^), Albumin, Alanine Aminotransferase (ALT), Aspartate Aminotransferase (AST), Blood Urea Nitrogen (BUN), Serum Uric Acid (UA), Estimated Glomerular Filtration Rate (eGFR), Total Cholesterol (TC), Triglycerides (TG), Lipoprotein (a) (Lp (a), Apolipoprotein B (apoB), Serum Calcium (CA), Fasting Plasma Glucose (FPG), and the Systemic Immune-Inflammation Index (SII). Comorbidities (including hypertension and diabetes) were defined based on patients’ clear prior hospital diagnostic history or medication records at the time of admission/examination. Routine laboratory assays were performed using standard automated biochemical analyzers in our hospital laboratory.

Core Exposure Variable: The core exposure variable in this study was Risk-weighted apolipoprotein B (RW-apoB). The calculation formula is: RW-apoB = 11.65 × TG (mmol/L) + 0.215 × Lp (a) (nmol/L) + 0.736 × apoB (mg/dL) (16). Based on the distribution characteristics of this index within the entire cohort, we stratified all subjects into three groups according to the tertiles of their RW-apoB levels: the low-level group (20.21–78.48), the medium-level group (78.48–99.50), and the high-level group (99.50–199.88),.in order to evaluate the association under different exposure burdens.

### 2.3 Statistical analysis

All statistical analyses were performed using R software. All statistical tests were two-sided, and a *P*-value < 0.05 was considered to indicate statistical significance.

Baseline Characteristics Description: Continuous variables were first tested for normality. Normally distributed continuous variables are expressed as mean ± standard deviation (Mean ± SD), and inter-group comparisons were performed using the independent samples t-test; non-normally distributed continuous variables are presented as the median (interquartile range, IQR), with inter-group comparisons conducted using the Mann–Whitney U test. Categorical variables are expressed as frequencies and percentages [*n* (%)], and comparisons between groups were performed using the Chi-square test or Fisher's exact test.

Multivariate Regression and Subgroup Stratification Analysis: To ensure the robustness of our models, participants with missing data for key variables were excluded via complete case analysis, given that the missingness mechanism was assumed to be at random and the proportion was negligible. Prior to modeling, multicollinearity was assessed using Variance Inflation Factors (VIF); all covariates demonstrated a VIF < 5, indicating no significant collinearity. Furthermore, with 239 events in the case group and fewer than 20 covariates, the events-per-parameter (EPP) ratio exceeded 10, satisfying the requirements for stable model specification. Binary Logistic Regression models were used to evaluate the association between RW-apoB levels and the prevalence of kidney stones, calculating the odds ratios (OR) and 95% confidence intervals (CI). We constructed four progressively adjusted regression models: Model 1 was a crude model without adjusting for any covariates; Model 2 was adjusted for age, gender, and BMI; Model 3 further adjusted for all clinical covariates listed in Table 1 (excluding the stratification variable itself); Because precise data regarding explicit lipid-lowering medication use (e.g., statins) were unavailable, we performed a sensitivity analysis (Model 4) to rigorously address this unmeasured confounding. Model 4 adjusted for the same covariates as Model 3 but strictly excluded all individuals with comorbid hypertension and diabetes. Given that these populations are the primary candidates for lipid-modifying therapy according to clinical guidelines, this restricted cohort represents individuals least likely to be receiving such treatments. Furthermore, to verify the stability of the study conclusions across populations with different characteristics, we conducted stratified subgroup analyses according to age (20–54 years, 55–85 years), gender (male, female), presence of hypertension (yes, no), and presence of diabetes (yes, no). To evaluate potential effect modification, formal interaction tests were conducted by including cross-product terms between RW-apoB and the stratification variables in the models. Additionally, linear trends across RW-apoB categories were tested by treating the median value of each tertile as a continuous variable (*P* for trend). Finally, to evaluate the clinical utility of the composite RW-apoB index, we performed a component analysis. This involved assessing the correlations between RW-apoB and its individual components (ApoB, TG, and Lp (a), and constructing a comparative multivariable logistic regression model that incorporated these three components simultaneously to compare their individual associations against the composite index.

**Table 1:** Baselines characteristics of participants.

Characteristic	Non-stone formers	Stone formers	*P*-value
*N*	522	239	
Age (years)	50.62 ± 12.52	51.05 ± 11.67	0.658
BMI	24.39 ± 3.74	24.50 ± 3.37	0.708
Albumin (g/L)	44.17 ± 4.05	43.78 ± 3.80	0.214
ALT (U/L)	25.45 ± 22.63	26.96 ± 24.28	0.404
AST (U/L)	22.00 ± 12.86	21.87 ± 12.95	0.896
BUN (mmol/L)	5.84 ± 3.27	5.98 ± 2.56	0.575
UA (μmol/L)	326.41 ± 89.10	344.19 ± 98.99	0.014
eGFR (mL/min/1.73 m^2^)	94.13 ± 24.81	87.30 ± 25.86	<0.001
TC (mmol/L)	4.55 ± 0.91	4.66 ± 0.97	0.131
CA (mmol/L)	2.34 ± 0.11	2.36 ± 0.16	0.982
FPG (mmol/L)	5.46 ± 1.41	5.29 ± 0.96	0.101
SII	531.65 ± 475.78	556.76 ± 805.24	0.592
RW-apoB	88.52 ± 24.42	95.89 ± 28.80	<0.001
Gender (%)			0.428
Male	303 (58.05%)	146 (61.09%)	
Female	219 (41.95%)	93 (38.91%)	
Diabetes (%)			0.26
No	426 (81.61%)	203 (84.94%)	
Yes	96 (18.39%)	36 (15.06%)	
High Blood Pressure (%)			0.777
No	355 (68.01%)	165 (69.04%)	
Yes	167 (31.99%)	74 (30.96%)	

Dose-Response Relationship: To observe the dynamic impact of continuous numerical changes in RW-apoB on the association with kidney stone prevalence, we employed a Generalized Additive Model (GAM) with penalized smoothing splines to plot dose-response curves. This approach visually and intuitively evaluates the potential linear and non-linear associations between continuously varying RW-apoB and prevalent kidney stones while avoiding arbitrary knot selection. Finally, to evaluate and compare the discriminatory efficacy of different lipid-related indices, we constructed receiver operating characteristic (ROC) curves as an exploratory analysis. The area under the curve (AUC) was calculated separately for risk-weighted apolipoprotein B (RW-apoB), apolipoprotein B (APOB) alone, triglycerides (TG), and lipoprotein (a) [Lp (a)], in order to quantify the comprehensive discriminatory ability of each index in distinguishing between stone patients and non-stone populations.

## 3 Results

### 3.1 Comparison of baseline characteristics of the study subjects

This study included a total of 761 subjects, comprising 522 in the non-stone control group and 239 in the kidney stone group. The baseline clinical and biochemical characteristics of the two groups are presented in Table 1. The analysis revealed no statistically significant differences between the kidney stone group and the non-stone group regarding age (51.05 ± 11.67 vs. 50.62 ± 12.52 years, *P* = 0.658), BMI (24.50 ± 3.37 vs. 24.39 ± 3.74, *P* = 0.708), gender distribution (male: 61.09% vs. 58.05%, *P* = 0.428), and the prevalence of hypertension (30.96% vs. 31.99%, *P* = 0.777) and diabetes (15.06% vs. 18.39%, *P* = 0.260).

Regarding laboratory biochemical indicators, the serum uric acid (UA) level in the kidney stone group was significantly higher than that in the non-stone group (344.19 ± 98.99 vs. 326.41 ± 89.10 μmol/L, *P* = 0.014), while the estimated glomerular filtration rate (eGFR) was significantly lower than in the non-stone group (87.30 ± 25.86 vs. 94.13 ± 24.81 mL/min/1.73 m^2^, *P* < 0.001). Concurrently, the RW-apoB level in the kidney stone group was significantly higher than that in the non-stone group (95.89 ± 28.80 vs. 88.52 ± 24.42, *P* < 0.001). Other indicators, including Albumin, ALT, AST, BUN, TC, CA, FPG, and SII, showed no significant statistical differences between the two groups.

### 3.2 Logistic regression and smooth curve fitting analysis of RW-apoB and kidney stone prevalence

To evaluate the association between RW-apoB and prevalent kidney stones, we constructed multivariate Logistic regression models (Table 2). When RW-apoB was included in the model as a continuous variable, the OR values for RW-apoB in Model 1, Model 2, and Model 3 were all 1.01 (95% CI: 1.00–1.02). In the sensitivity analysis excluding individuals with hypertension and diabetes (Model 4), the positive association remained consistent (OR = 1.01, 95% CI: 1.00–1.03). To better quantify this continuous effect and assess clinical relevance, we further calculated the adjusted risk per 10-unit and per standard deviation (1-SD) increase in RW-apoB. In the sensitivity model (Model 4), every 10-unit increase in RW-apoB was associated with an OR of 1.15 (95% CI: 1.00–1.31), and every 1-SD increase was associated with an OR of 1.40 (95% CI: 1.01–1.93).

**Table 2:** Logistic regression analysis between RW-apoB with kidney stones prevalence.

Characteristic	Model 1 OR (95% CI)	Model 2 OR (95% CI)	Model 3 OR (95% CI)	Model 4 OR (95% CI)
RW-apoB	1.01 (1.00, 1.02)	1.01 (1.00, 1.02)	1.01 (1.00, 1.02)	1.01 (1.00, 1.03)
Per 10 units	1.11 (1.05, 1.18)	1.12 (1.05, 1.19)	1.17 (1.07, 1.27)	1.15 (1.00, 1.31)
RW-apoB-SD	1.32 (1.13, 1.54)	1.34 (1.14, 1.57)	1.50 (1.20, 1.87)	1.40 (1.01, 1.93)
Categories				
Low (20.21–78.48)	1	1	1	1
Middle (78.48–99.50)	0.95 (0.64, 1.40)	0.96 (0.65, 1.42)	0.89 (0.58, 1.37)	1.31 (0.76, 2.26)
High (99.50–199.88)	1.51 (1.04, 2.18)	1.53 (1.04, 2.25)	1.43 (0.85, 2.38)	1.40 (0.68, 2.86)
*P* for trend	0.03	0.03	0.18	0.56

Model 1 was adjusted for no covariates.

Model 2 was adjusted for age, gender and BMI.

Model 3 was adjusted for covariates in Table 1.

Model 4 was adjusted for the same variables as Model 3 and excluded individuals with hypertension and diabetes.

When RW-apoB was analyzed categorically according to tertiles (with the low-level group as the reference):

Medium-level group (78.48–99.50): In the unadjusted model (Model 1: OR = 0.95, 95% CI: 0.64–1.40), the demographic-adjusted model (Model 2: OR = 0.96, 95% CI: 0.65–1.42), and the multivariable-adjusted model (Model 3: OR = 0.89, 95% CI: 0.58–1.37), the 95% confidence intervals crossed 1.

High-level group (99.50–199.88): In Model 1 (OR = 1.51, 95% CI: 1.04–2.18) and Model 2 (OR = 1.53, 95% CI: 1.04–2.25), the high-level group demonstrated a significantly increased association with kidney stones. However, in Model 3, which comprehensively adjusted for all clinical covariates in Table 1, the OR was 1.43 (95% CI: 0.85–2.38), and the confidence interval crossed 1. Ultimately, in the strict sensitivity analysis (Model 4), the risk estimates for the medium- and high-level groups were 1.31 (95% CI: 0.76–2.26) and 1.40 (95% CI: 0.68–2.86), respectively. Furthermore, we performed a trend test across the RW-apoB tertiles. The *P* for trend was statistically significant in the crude and demographically adjusted models (*P* = 0.03 for Models 1 and 2), but lost significance in the fully adjusted Model 3 (*P* = 0.18) and the sensitivity Model 4 (*P* = 0.56). This indicates that while the continuous evaluation of RW-apoB maintains robust statistical power, categorizing the exposure into discrete tertiles reduces the statistical efficiency and compromises significance in heavily adjusted models.

To visually explore the potential morphological association between continuous RW-apoB and prevalent kidney stones, we constructed a multiply-adjusted Restricted Cubic Spline (RCS) model. After adjusting for potential clinical confounding variables (Model 3), the smooth curve exhibited an overall positive correlation trend (Figure 2). As the RW-apoB level increased within the exposure range, the probability of prevalent kidney stones demonstrated a general monotonic increase. To further justify the clinical utility of the composite RW-apoB index over its individual parts, we evaluated its mathematical components (ApoB, TG, and Lp (a). The correlation scatter plots between RW-apoB and each individual component are presented in Supplementary Figure S1. Furthermore, we constructed a comparative logistic regression model evaluating these three components individually (Supplementary Table S1). In the fully adjusted multivariable model (Model 3), the individual associations for ApoB (OR = 1.03, 95% CI: 0.98–1.08) and TG (OR = 1.01, 95% CI: 0.92–1.11) lost statistical significance, leaving only Lp (a) (OR = 1.35, 95% CI: 1.04–1.77) as a significant independent factor. Conversely, the composite RW-apoB index maintained a stable and consistent positive association with prevalent kidney stones in our main analysis. This suggests that evaluating these lipid parameters separately may fragment the assessment of total pathogenic lipid burden, whereas the mathematically weighted composite RW-apoB serves as a more stable and comprehensive proxy.

**Figure 2:**
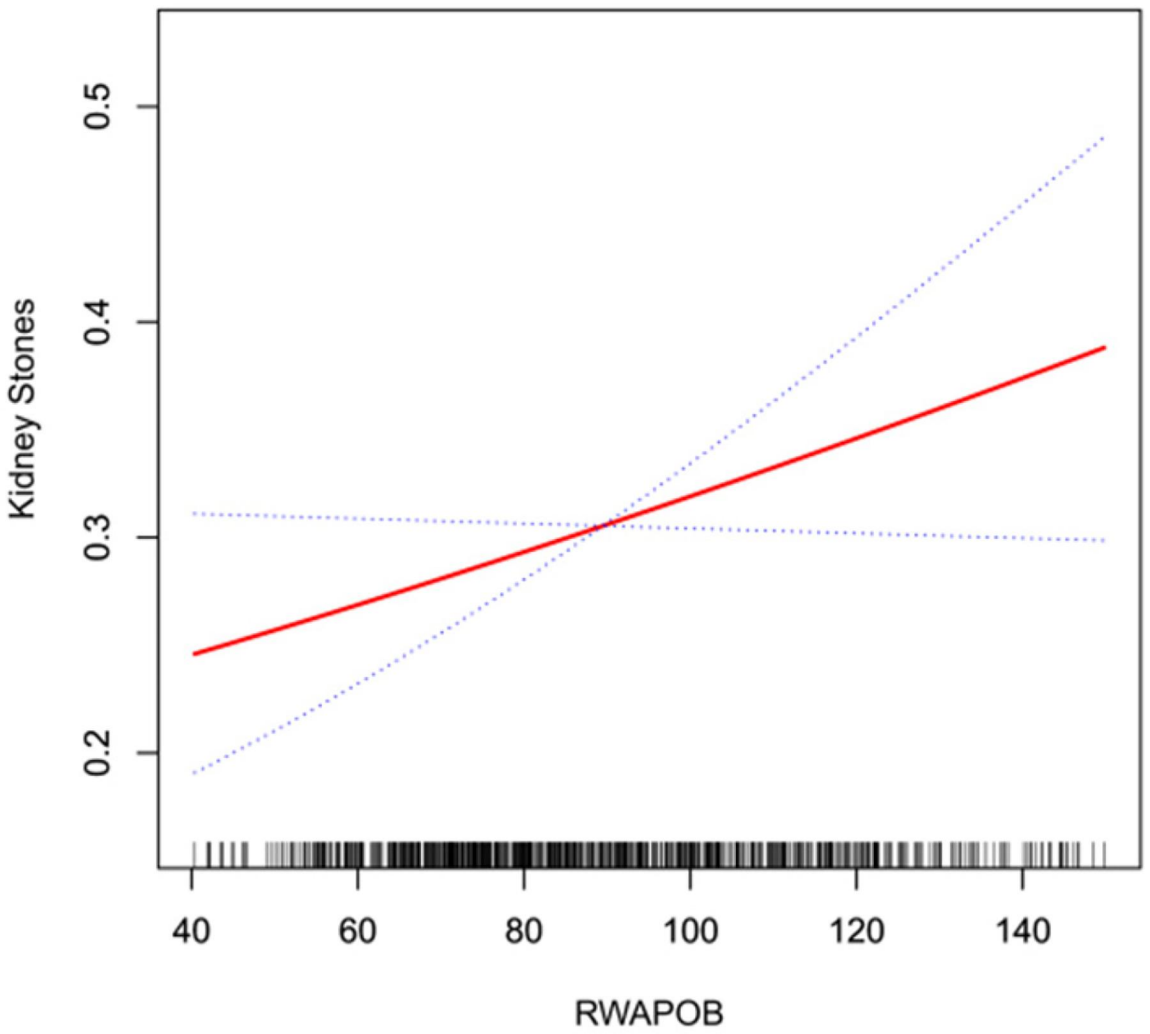
Generalized additive model (GAM) plot representing the dose-response relationship between continuous RW-apoB levels and prevalent kidney stones. The solid red line represents the estimated association, and the dotted blue lines represent the 95% confidence bands. The data density is displayed via the rug plot on the *x*-axis. The model utilized penalized smoothing splines adjusted for covariates.

### 3.3 Stratified subgroup analysis

To verify the stability of the aforementioned results across populations with different characteristics, we performed stratified regression analyses according to age, gender, hypertension, and diabetes (Table 3). The results demonstrated that RW-apoB, as a continuous variable, maintained a relatively consistent positive trend with prevalent kidney stones across all clinical subgroups. In Model 3, which adjusted for all laboratory indicators, the OR values of each subgroup remained stable between 1.01 and 1.02. Among them, in the female population (OR = 1.02, 95% CI: 1.01–1.03) and the population with comorbid hypertension (OR = 1.02, 95% CI: 1.01–1.03), the confidence intervals were completely above 1, demonstrating clear statistical significance. Conversely, in the 55–85 age group (OR = 1.01, 95% CI: 1.00–1.04) and the diabetic population (OR = 1.02, 95% CI: 1.00–1.04), the confidence intervals touched the boundary of 1, indicating a positive correlation trend. Crucially, formal interaction tests revealed no significant effect modification across any of these strata (all *P* for interaction > 0.05, specifically: age *P* = 0.75, gender *P* = 0.16, hypertension *P* = 0.18, diabetes *P* = 0.39). This mathematically demonstrates that the positive association between continuous RW-apoB and kidney stones is broadly uniform regardless of age, gender, or metabolic comorbidities.

**Table 3:** Stratified analysis between RW-apoB with kidney stones prevalence.

Characteristic	Model 1 OR (95% CI)	Model 2 OR (95% CI)	Model 3 OR (95% CI)	*P* for interaction
Stratified by age (years)				0.75
20–54	1.01 (1.00, 1.02)	1.01 (1.00, 1.02)	1.01 (1.00, 1.02)	
55–85	1.01 (1.00, 1.02)	1.01 (1.00, 1.02)	1.01 (1.00, 1.04)	
Stratified by gender				0.16
Male	1.01 (1.00, 1.02)	1.01 (1.00, 1.02)	1.01 (1.00, 1.02)	
Female	1.02 (1.01, 1.03)	1.02 (1.01, 1.03)	1.02 (1.01, 1.03)	
Stratified by hypertension				0.18
No	1.01 (1.00, 1.02)	1.01 (1.00, 1.02)	1.01 (1.00, 1.03)	
Yes	1.02 (1.01, 1.03)	1.02 (1.01, 1.03)	1.02 (1.01, 1.03)	
Stratified by diabetes				0.39
No	1.01 (1.00, 1.02)	1.01 (1.00, 1.02)	1.01 (1.00, 1.02)	
Yes	1.02 (1.00, 1.03)	1.02 (1.00, 1.03)	1.02 (1.00, 1.04)	

Model 1 was adjusted for no covariates.

Model 2 was adjusted for age, gender and BMI.

Model 3 has been adjusted for the covariates listed in Table 1, excluding the categorical variables themselves.

Because precise data regarding explicit lipid-lowering medication use (e.g., statins) were unavailable, we performed a sensitivity analysis (Model 4) to rigorously address this unmeasured confounding. Model 4 adjusted for the same covariates as Model 3 but strictly excluded all individuals with comorbid hypertension and diabetes. Given that these populations are the primary candidates for lipid-modifying therapy according to clinical guidelines, this restricted cohort represents individuals least likely to be receiving such treatments.

### 3.4 Exploratory ROC curve analysis

To explore the potential discriminatory ability of RW-apoB for prevalent kidney stones, we conducted a receiver operating characteristic (ROC) curve analysis as an exploratory evaluation compared to traditional lipid indices (Figure 3 and Supplementary Table S2). The analysis revealed that the area under the curve (AUC) for RW-apoB was 0.561 (95% CI: 0.516–0.606). In comparison, the AUC for apoB alone was 0.553 (95% CI: 0.508–0.598), lipoprotein (a) [Lp (a)] was 0.499 (95% CI: 0.453–0.544), and triglycerides (TG) was 0.483 (95% CI: 0.438–0.527). A formal pairwise comparison between the AUCs of RW-apoB and apoB revealed no statistically significant difference (*P* > 0.05). Consistent with the wide confidence intervals overlapping the chance level (0.5), these findings indicate that while RW-apoB maintains a positive epidemiological association with kidney stones in regression models, it possesses weak discriminatory ability and serves strictly as an associative biomarker in this retrospective setting, rather than a standalone clinical diagnostic tool.

**Figure 3:**
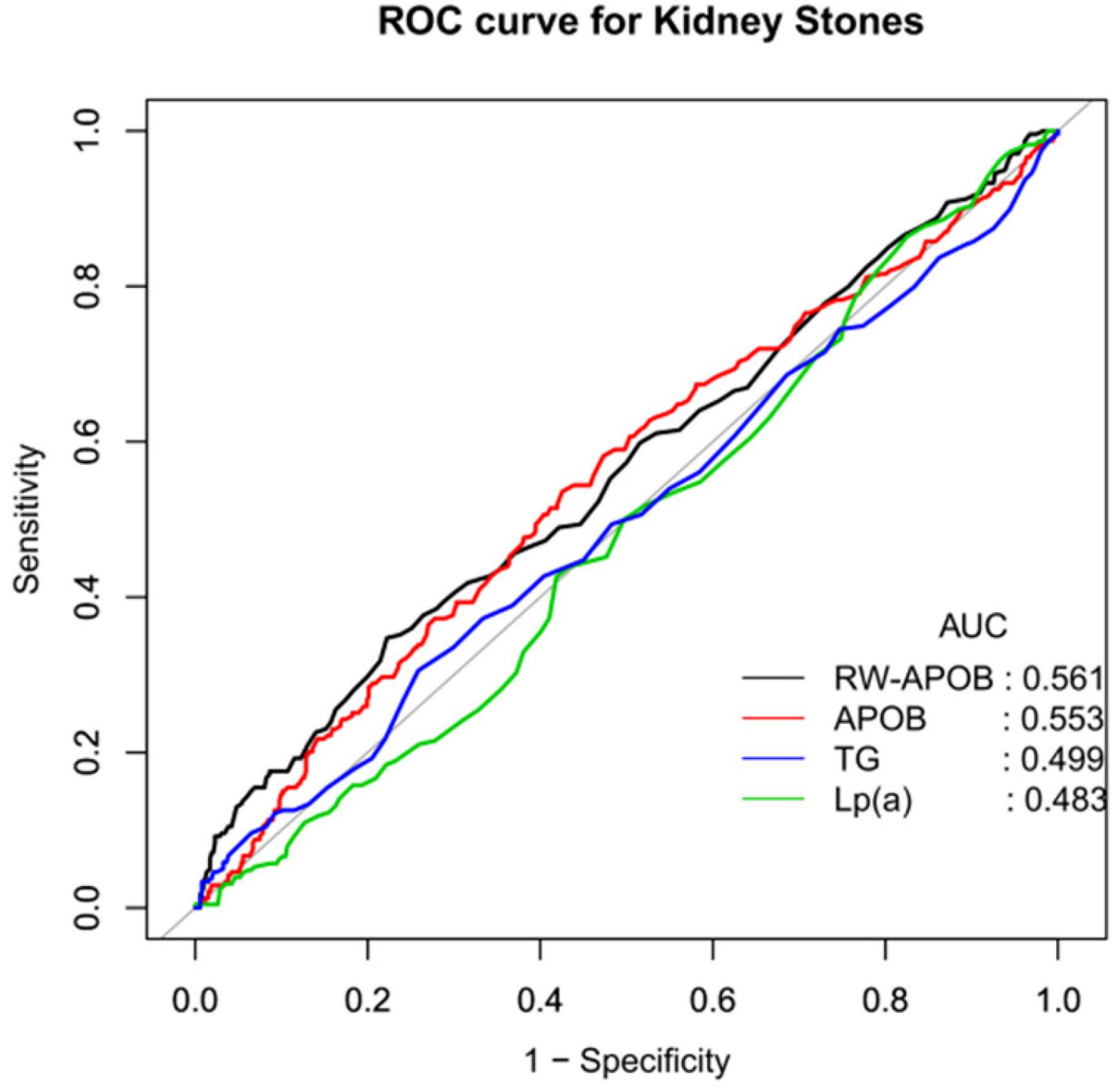
Receiver operating characteristic (ROC) curves of different lipid-related indices for predicting the risk of kidney stones.

## 4 Discussion

This study explores the association between risk-weighted apolipoprotein B (RW-apoB) levels and prevalent kidney stones. Our primary finding is that plasma RW-apoB levels in patients with incident kidney stones were significantly higher than those in the non-stone control population. In our multivariable regression analysis, continuously elevated RW-apoB was positively associated with prevalent kidney stones. Importantly, this positive association remained consistent in our rigorous sensitivity analysis that excluded individuals with comorbid hypertension and diabetes, effectively addressing the unmeasured confounding of lipid-lowering therapies. Furthermore, the Restricted Cubic Spline (RCS) analysis demonstrated a generally positive correlative trend as continuous RW-apoB levels increased. Our stratified analysis indicated that this association remained relatively stable across various clinical subgroups.

The results of this study corroborate that systemic lipid abnormalities are closely associated with urinary stone prevalence. Previous large-scale epidemiological studies have confirmed that traditional lipid components (such as elevated triglycerides, elevated low-density lipoprotein cholesterol, and decreased high-density lipoprotein) are closely related to prevalent kidney stones (9, 21, 22). However, because each atherogenic lipoprotein particle contains only one apoB molecule, apoB-based indices are far superior to traditional blood lipid concentration indices in quantifying the total burden of pathogenic lipids (13, 23). Large cohort studies in the cardiovascular and metabolic fields have fully demonstrated that apoB and derived composite indices (such as RW-apoB) can more precisely capture residual metabolic risk (16). It is important to note that RW-apoB was originally developed specifically for coronary-heart-disease prediction (16), and subsequent discussions have highlighted considerations regarding its mathematical dependency and transportability to other disease contexts (24). However, exploring lipid markers in renal contexts remains highly relevant. Recent studies have demonstrated significant associations between remnant cholesterol and nephrolithiasis (25, 26). Furthermore, broader renal literature underscores the critical role of dyslipidemia in chronic kidney disease (CKD), such as the impact of remnant cholesterol on CKD progression (27) and general dyslipidemia in CKD (28). Our findings align with this emerging body of evidence, suggesting that composite apoB-based indices may similarly reflect metabolic derangements associated with kidney stones. In our study, even after adjusting for multiple traditional risk factors, a high RW-apoB burden remained significantly associated with prevalent kidney stones. More importantly, our subgroup analysis showed that this association was particularly pronounced in the female population and the population with comorbid hypertension. On one hand, during the perimenopausal and postmenopausal periods, females experience a deterioration in their lipid metabolism profile due to a sharp decline in estrogen levels, which normally provide renal and metabolic protection; renal tubular epithelial cells lacking estrogen protection are more sensitive to crystal adhesion caused by lipid peroxidation, thereby amplifying the promoting effect of lipid burden on stone formation (29, 30). On the other hand, hypertension itself is a hallmark of systemic endothelial dysfunction (31, 32). In classic cardiovascular and metabolic pathological mechanisms, an impaired vascular endothelial barrier is the initiating factor that leads to the abnormal penetration and retention of apoB-rich atherogenic lipoproteins in the subintima (33–35). Simultaneously, vascular damage in the renal papillary microcirculation leads to localized ischemia and interstitial calcification, which is considered a core early event triggering stone formation (36). Therefore, we hypothesize that the compromised endothelial barrier caused by hypertension provides a perfect penetration target for high-burden RW-apoB; the “two-hit” of both factors produces a profound synergistic destructive effect, exacerbating local renal lipotoxicity and oxidative stress, thereby accelerating the early calcification of the stone attachment base (Randall's plaques). This provides a mechanistic hypothesis for the significantly greater stone susceptibility to elevated RW-apoB observed in hypertensive patients in our study.

The potential mechanisms by which high levels of RW-apoB promote kidney stone formation may involve multiple intertwining pathological pathways. First, lipotoxicity-mediated renal tubular injury and oxidative stress serve as a critical hub for stone formation. High-burden pathogenic lipoproteins not only trigger systemic inflammation, but their excessive deposition in renal tubular cells leads to lipotoxicity and even ferroptosis, triggering a massive release of reactive oxygen species (ROS) (12, 37). The damaged renal tubular epithelial cell membranes expose large quantities of adhesion molecules (such as Osteopontin, CD44, and hyaluronic acid), causing microcrystals that would otherwise be excreted in urine to become firmly adhered and retained within the kidneys (38, 39). Second, urine acidification driven by insulin resistance (IR) plays a central role. High apoB levels are a strong predictor of systemic insulin resistance (40). IR directly impairs the ability of the proximal renal tubules to produce and secrete ammonia (NH3/NH4+), leading to an abnormal decrease in urine pH; this acidic microenvironment drastically reduces the solubility of uric acid and is the strongest biochemical driver for inducing uric acid stones and mixed stones (41–43). Finally, the Vascular Theory and Randall's Plaques provide a morphological explanation for lipid-induced lithogenesis. Similar to the pathological process of atherosclerosis, the deposition of highly pathogenic lipids in the renal capillary network causes local chronic ischemia and inflammatory infiltration, inducing dystrophic calcification in the renal papillary interstitium—this is precisely the initiating step in the formation of Randall's plaques (i.e., the “hotbed” for calcium oxalate stone growth) (36, 44, 45).

Objectively speaking, while this study holds certain innovativeness and clinical significance, it also has inherent limitations. The primary strength of this study lies in it being the first internationally to introduce an advanced composite lipid index (RW-apoB) from the forefront of cardiovascular and metabolic fields into kidney stone research, broadening the metabolic perspective of stone etiology. However, there are shortcomings: First, as a single-center retrospective case-control study, the study design cannot definitively determine the deterministic causal temporal relationship between elevated RW-apoB and stone occurrence; multicenter prospective cohort studies are urgently needed for validation in the future. Second, limited by the electronic medical record system, we were unable to obtain detailed patient dietary habit data (such as fluid, sodium, and animal protein intake), which have been proven to be important independent variables affecting stone pathogenesis. Third, this study lacks comprehensive detection data on 24-hour urine metabolic components (such as 24 h urinary calcium, urinary oxalate, urinary citrate), failing to directly assess the specific impact of RW-apoB on the physical and chemical saturation of urinary lithogenesis. Fourth, the cases and controls were drawn from different sampling frames—hospitalized symptomatic urology patients versus a health-examination population. This introduces inherent selection bias, as individuals undergoing routine health check-ups may represent a more health-conscious demographic with fundamentally different baseline metabolic and behavioral profiles, potentially limiting the direct comparability of the two source populations.

## 5 Conclusion

Higher RW-apoB was associated with prevalent kidney-stone status after adjustment for measured clinical covariates.

## Data Availability

The raw data supporting the conclusions of this article will be made available by the authors, without undue reservation.
