# Supplementary material for: Association between risk-weighted apolipoprotein B and prevalent kidney stones: a retrospective case-control study
Source: Front Med (Lausanne). 2026 Sep 14;13:1927930. doi: 10.3389/fmed.2026.1927930 (PMC13616615; doi:10.3389/fmed.2026.1927930)
Supplement: Supplementary file 1 [file Table1.docx]

Supplementary table 1. Logistic regression analysis between different index with kidney stones.

| **Characteristic** | **Model 1 OR (95% CI)** | **Model 2 OR (95% CI)** | **Model 3 OR (95% CI)** |
| --- | --- | --- | --- |
| ApoB | 0.87 (0.84, 0.90) | 1.01 (0.97, 1.05) | 1.03 (0.98, 1.08) |
| TG | 1.32 (1.22, 1.42) | 1.09 (1.00, 1.18) | 1.01 (0.92, 1.11) |
| LP(a) | 0.19 (0.13, 0.26) | 1.24 (0.99, 1.55) | 1.35 (1.04, 1.77) |

Supplementary table 2. Receiver operating characteristic (ROC) curve analysis of different lipid-related indices for prevalent kidney stones.

| Test | Yes | No | ROC area(AUC) | 95%CI low | 95%CI upp | Best threshold | Specificity | Sensitivity |
| --- | --- | --- | --- | --- | --- | --- | --- | --- |
| RW-ApoB | 239 | 522 | 0.5612 | 0.5163 | 0.6061 | 0.965 | 0.7778 | 0.3473 |
| ApoB | 239 | 522 | 0.5529 | 0.5082 | 0.5975 | 1.515 | 0.5747 | 0.5356 |
| TG | 228 | 497 | 0.4826 | 0.4383 | 0.5269 | 0.185 | 0.1751 | 0.864 |
| LP(a) | 239 | 522 | 0.4985 | 0.4532 | 0.5439 | 2.405 | 0.7414 | 0.3054 |
